# Inertia-driven resonant excitation of a magnetic skyrmion

**DOI:** 10.1038/s41598-017-13241-2

**Published:** 2017-10-25

**Authors:** Takayuki Shiino, Kab-Jin Kim, Ki-Suk Lee, Byong-Guk Park

**Affiliations:** 1 Department of Materials Science and Engineering, KAIST, Daejeon, 34141 Republic of Korea; 2 Department of Physics, KAIST, Daejeon, 34141 Republic of Korea; 3 School of Materials Science and Engineering, Ulsan National Institute of Science and Technology, Ulsan, 689-798 Republic of Korea

## Abstract

Topological spin structures such as magnetic domain walls, vortices, and skyrmions, have been receiving great interest because of their high potential application in various spintronic devices. To utilize them in the future spintronic devices, it is first necessary to understand the dynamics of the topological spin structures. Since inertial effect plays a crucial role in the dynamics of a particle, understanding the inertial effect of topological spin structures is an important task. Here, we report that a strong inertial effect appears steadily when a skyrmion is driven by an oscillating spin-Hall-spin-torque (SHST). We find that the skyrmion exhibits an inertia-driven hypocycloid-type trajectory when it is excited by the oscillating SHST. This motion has not been achieved by an oscillating magnetic field, which only excites the breathing mode without the inertial effect. The distinct inertial effect can be explained in terms of a spin wave excitation in the skyrmion boundary which is induced by the non-uniform SHST. Furthermore, the inertia-driven resonant excitation provides a way of experimentally estimating the inertial mass of the skyrmion. Our results therefore pave the way for the development of skyrmion-based device applications.

## Introduction

The inertial effect of a topological spin structure is one of the long-standing unresolved issues in magnetism^1–7^. Since the inertial effect originates from the deformation of internal spin structure^1,2^, it generally appears in transient states, i.e., at the onset or after the removal of an external force, and produces an additional displacement due to an inertia-driven acceleration or deceleration effect^3–5^. For instance, a magnetic domain wall (DW) exhibits an inertial effect due to the deformation of internal spin structure^1,6^. The consequent modification of the DW motion makes it difficult to precisely control the DW position^3–5^. Furthermore, a DW shows a resonant behaviour when it experiences an oscillating external force, and this allows one to determine the mass of the DW based on the simple harmonic oscillator approach^7^. An outstanding question is whether such an inertial effect appears in other topological spin structures.

Recently, magnetic skyrmions^8–12^, which are topologically protected winding spin structures, have received a great deal of attention due to their possible application to future spintronic devices^13–17^. Magnetic skyrmions can be created at room temperature through the chiral interaction known as the Dzyaloshinskii-Moriya interaction (DMI)^18–23^. Considering that the skyrmion contains a magnetic DW at its circular boundary, it is expected that the skyrmion can also exhibit an inertial effect when it moves. Recent studies have indeed observed the inertial effect of the skyrmion during the relaxation process^24^. When the skyrmion is shifted from its equilibrium position, the spin structures in the circular boundary of the skyrmion, i.e., the spins inside the DW, are generally deformed. When the skyrmion is relaxed, the deformed spins are released through spin wave propagation along the DW channel. This additional degree of freedom originating from the in-plane magnetization of the circular boundary of the skyrmion yields an inertial effect on the skyrmion and generates a unique motion, that is, a hypocycloid-type motion^2,25,26^.

So far, this inertia-driven hypocycloid-type motion of the skyrmion has only been observed during the relaxation process^25,26^. Unlike magnetic DWs, inertia-driven resonant excitation by an oscillating external field has not been observed in skyrmions, because the oscillating field generates either a gyrotropic motion or a breathing motion in the skyrmion without deforming the boundary spin structure. Here we report our discovery that inertia-driven resonant excitation can be achieved in the skyrmion by employing an oscillating spin-Hall-spin-torque (SHST).

## Results

### Model

**Figure 1:**
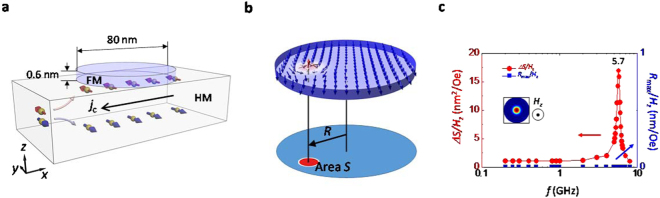
Skyrmion in a magnetic nanodisk and its excitation by magnetic field. (**a**) Illustration of a HM/FM magnetic nanodisk system with electrons flowing in the HM electrode. The diameter and the thickness of the FM disk were set to 80 nm and 0.6 nm, respectively. (**b**) Illustration of a skyrmion confined in the nanodisk: skyrmion position *R* from the disk centre and the area of the skyrmion *S*. The colour indicates the magnitude of *m*_*z*_: red for *m*_*z*_ = +1 and blue for *m*_*z*_ = −1. (**c**) Frequency spectrum of the *R*_max_ (blue: right axis) and *ΔS* (red: left axis) under an oscillating out-of-plane magnetic field. Note that *R*_max_ and *ΔS* are normalized by the amplitude *H*_*z*_.

To explore the inertia-driven resonant excitation of the skyrmion, we model an interface-induced skyrmion in a ferromagnet (FM)/nonmagnet (NM) bi-layer nanodisk with a diameter of 80 nm. In the simulation, the skyrmion has a Neel-type DW at its boundary due to the interfacial DMI, which inherently exists at the interface between the FM and the NM layers (see Fig. 1a). We excite the skyrmion by applying an oscillating field or current of various frequencies. The excitation mode of the skyrmion can be characterised by the skyrmion position **R** = (*X*, *Y*) from the disk centre, $R=|{\bf{R}}|=\sqrt{{X}^{2}+{Y}^{2}}$, and the area of the skyrmion *S*, as shown in Fig. 1b. In this definition, the increase in the maximum displacement *R*_max_ and the area variation *ΔS* in the frequency spectrum correspond to the gyrotropic mode excitation and the breathing mode excitation, respectively.

### Breathing mode of a skyrmion induced by an oscillating out-of-plane magnetic field

We first examine the magnetic-field-driven resonant excitation of the skyrmion. To this end, we first locate the skyrmion at the disk centre and then apply an oscillating out-of-plane magnetic field, ${\bf{H}}={{\bf{e}}}_{z}{H}_{z}\,\sin (2\pi {f}_{{\rm{a}}{\rm{c}}}^{H}t)$, with various frequencies ${f}_{{\rm{ac}}}^{H}$. Here ${H}_{z}=50\,{\rm{Oe}}$ $(5.5\,{\rm{G}}{\rm{H}}{\rm{z}}\le {f}_{{\rm{a}}{\rm{c}}}^{H}\le 5.9\,{\rm{G}}{\rm{H}}{\rm{z}})$ and ${H}_{z}=100\,{\rm{Oe}}$ (elsewhere). Figure 1c shows the frequency spectrum of the *R*_max_ (blue) and *ΔS* (red), which are normalized by *H*_*z*_. While no significant variation is observed in the *R*_max_ spectrum, a clear peak is observed in the *ΔS* spectrum at ${f}_{{\rm{ac}}}^{H}\approx 5.7$ GHz, indicating that the breathing mode is excited at this frequency. Physically, the resonant excitation of the breathing mode originates from the resonant oscillation of the DW at the circular edge of the skyrmion. Therefore,$\,{f}_{{\rm{ac}}}^{H}\approx 5.7$ GHz corresponds to the resonance frequency of the DW, which is determined by the DW anisotropy energy potential, i.e., the energy difference between Neel- and Bloch-type DW’s^27^. It should be noted that there is no inertia-induced hypocycloid-type motion in this resonant breathing mode, indicating that the oscillating magnetic field does not deform the boundary spin structures of the skyrmion. That is to say, the phase of the DW oscillation exactly coincides with the resonant skyrmion breathing motion, so that there is no deformation of the spin structure inside the DW.

### Three skyrmion-mode excitations under oscillating SHST

**Figure 2:**
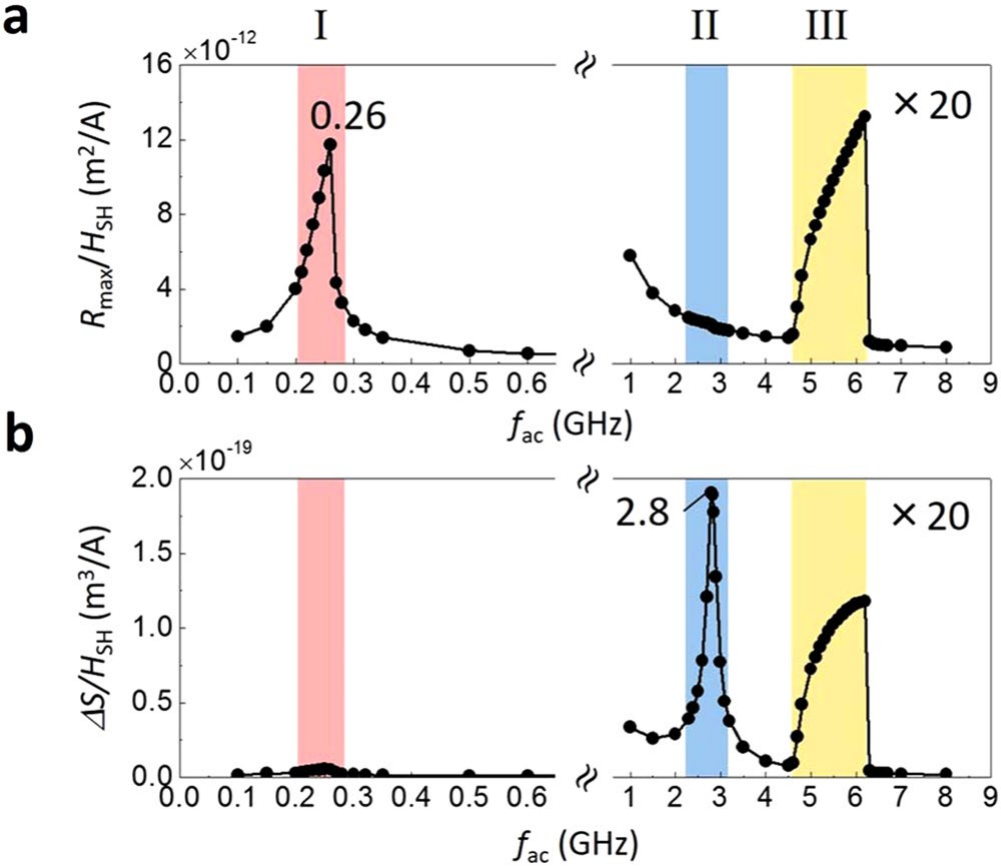
Frequency spectra of skyrmion mode excitations under an oscillating SHST. **(a**,**b**) Frequency spectra of (**a**) the maximum displacement *R*_max_ and (**b**) the area variation *ΔS*. Note that *R*_max_ and *ΔS* are normalized by the amplitude *H*_SH_, and they are amplified 20 times for ${f}_{{\rm{ac}}}\ge 1$ GHz. Regimes I,II, and III correspond to the gyrotropic mode (red-shaded), the breathing mode (blue-shaded), and the inertia-driven resonant excitation (yellow-shaded), respectively.

We next investigate the resonant excitation of the skyrmion driven by the spin Hall effect (SHE). We initially locate the skyrmion at the disk centre and then apply an in-plane AC current of frequency *f*_ac_ along the NM layer. Then the spins accumulated at the interface between the FM and the NM due to the SHE of the NM layer diffuse into the FM layer and exert an oscillating SHST to the skyrmion in the FM layer (see Methods). Figure 2 shows the frequency spectra of the skyrmion-mode excitations under the oscillating SHST. As we increase the *f*_ac_, three different excitation modes are observed. At a relatively low frequency regime, a sharp peak is observed in *R*_max_ at *f*_ac_ $\approx$ 0.26 GHz (regime I: the area shown in red). With a further increase in the *f*_ac_, we observe a sharp peak in *ΔS* at *f*_ac_ $\approx$ 2.8 GHz (regime II: the area shown in blue), and a broad peak both in *R*_max_ and *ΔS* at *f*_ac_ = 5 ~ 6 GHz (regime III: the area shown in yellow).

### Regime I: gyrotropic mode

**Figure 3:**
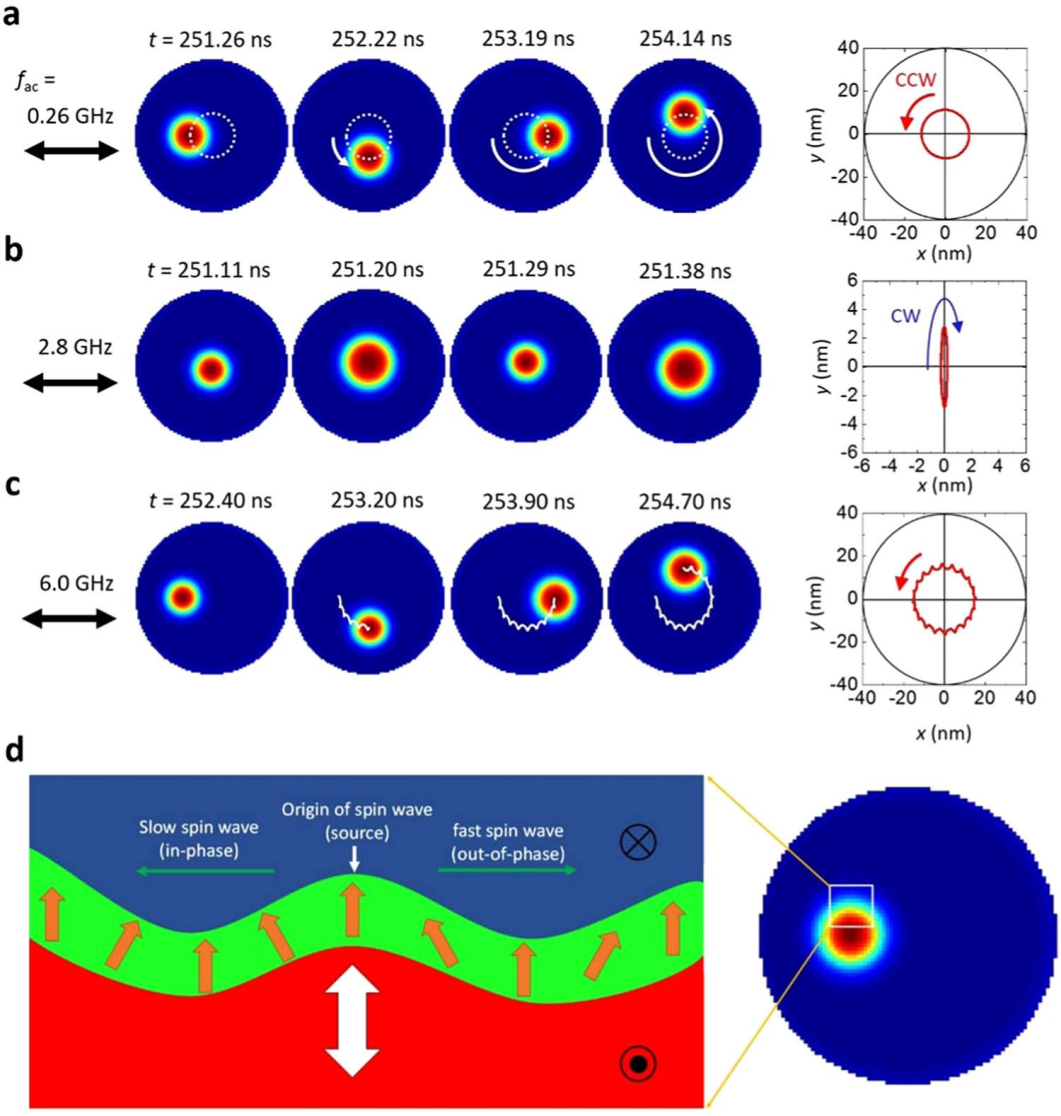
Snapshot images and trajectories of skyrmion-mode excitations induced by an oscillating SHST. **(a**,**b**,**c**), Snapshot images and the trajectories of a skyrmion under steady states for (**a**) the gyrotropic mode (0.26 GHz), (**b**) the breathing mode (2.8 GHz), and (**c**) the dual excitation mode of gyrotropic and breathing modes in the presence of strong inertial effects (6.0 GHz). The arrows in the trajectories indicate the directions of the skyrmion motion: counterclockwise (CCW) or clockwise (CW) rotations. (**d**) Illustration of excited spin waves propagating in opposite directions inside the DW channel.

In order to understand the observed frequency spectra, the trajectories of the skyrmion and corresponding snapshot images are extracted from the simulation and plotted in Fig. 3 (see also Supplementary Movies). It is clear that the resonant peak in *R*_max_ at *f*_ac_ $\approx$ 0.26 GHz corresponds to the gyrotropic motion (Fig. 3a), which is typically seen in two dimensional topological spin structures, such as vortices. This gyrotropic motion can be described by Thiele’s equation^28^ as

1 $${\bf{G}}\times \dot{{\bf{R}}}-k{\bf{R}}-D\dot{{\bf{R}}}=0,$$

where ${\bf{G}}=(0,0,G)$ is the gyrocoupling vector, $k$ is the effective spring constant induced by a geometrical potential, and *D* is the dissipation tensor, and $\dot{{\bf{R}}}$ is the time derivative of the position **R**. The gyrotropic motion of the skyrmion is analogous to a massless particle with an electrical charge in a magnetic field and an external potential through a viscous medium^15^. If we neglect the dissipation term, the resonance frequency, *f*_c_, can be easily calculated to be $2\pi {f}_{{\rm{c}}}=k/G$. Therefore, the resonant gyrotropic motion can be observed at ${f}_{{\rm{ac}}}={f}_{{\rm{c}}}$. This corresponds to the excitation mode observed in regime I. It should be noted that there is no inertial effect in regime I because the gyrotropic motion does not accompany the deformation of spin structures at the skyrmion boundary.

### Regime II: breathing mode

In regime II, on the other hand, a clear peak is observed in *ΔS* at *f*_ac_ $\approx$ 2.8 GHz, indicating the resonant excitation of the breathing mode, which is demonstrated in Fig. 3b. This breathing mode is similar to the field-driven resonant excitation as shown in Fig. 1c. However, the resonance frequency is exactly half of that for the field-driven case (${f}_{{\rm{ac}}}^{H}$ $\approx$ 5.7 GHz). Such a difference in resonance frequency originates from the confinement effect of the skyrmion in a circular disk. Unlike a magnetic field, the SHST generally induces a transverse motion in the skyrmion (which is known as the skyrmion Hall effect)^29^. As a result, the skyrmion centre moves along the transverse direction, generating an elliptical trajectory (see the trajectory in Fig. 3b). When the skyrmion deviates from the centre of the disk, it experiences a geometry-induced magnetic field whose frequency is twice that of the *f*_ac_ due to the elliptical trajectory. This is the reason why we observe a resonant breathing mode at ${f}_{{\rm{ac}}}=\frac{1}{2}{f}_{{\rm{ac}}}^{H}\approx 2.8$ GHz. This implies that the resonant breathing mode observed at ${f}_{{\rm{ac}}}\approx 2.8$ GHz can be understood as a resonant excitation by the geometry-induced field. Like the gyrotropic mode (regime I), the inertial effect does not appear in the resonant breathing mode.

### Regime III: inertia-driven hypocycloid-type mode

Contrary to regimes I and II, a broad peak is observed both in *R*_max_ and *ΔS* at ${f}_{{\rm{a}}{\rm{c}}}=5\sim 6$ GHz, which implies that the breathing mode and the gyrotropic mode are simultaneously excited. The corresponding skyrmion trajectory and snapshots are shown in Fig. 3c. This result is clearly distinguished from the magnetic-field-driven case in a similar frequency regime, where only the breathing mode is excited (Fig. 1c). The clear hypocycloid-type trajectory observed in regime III indicates that the inertia-driven resonant excitation of the skyrmion is achieved by the oscillating SHST.

According to a recent theory^2^, the inertial effect of a skyrmion can be described by inserting the Newtonian mass (*M*) term into Thiele’s equation as

2 $$-M\ddot{{\bf{R}}}+{\bf{G}}\times \dot{{\bf{R}}}-k{\bf{R}}-D\dot{{\bf{R}}}=0.$$

If we neglect the dissipation term (*D* = 0), Eq. (2) gives two circular modes with frequencies,

3 $${\omega }_{\pm }=-\frac{G}{2M}\pm \,\sqrt{{(\frac{G}{2M})}^{2}+\frac{k}{M}}$$

In the first order approximation with a small *M*, each frequency can be reduced to

4 $${\omega }_{+}=\frac{k}{G},\quad \,{\omega }_{-}=-\frac{G}{M}-\frac{k}{G}.$$

This indicates that the inertial mass simultaneously creates two circular modes with an opposite sign of frequency. One mode gives a counterclockwise (CCW) rotation with a relatively low frequency (${\omega }_{+}$), and the other mode gives a clockwise (CW) rotation with a relatively high frequency (${\omega }_{-}$). The combination of the two circular motions generates a hypocycloidal motion, which we observed in regime III. Note that the low-lying mode (${\omega }_{+}$) is identical to the resonant gyrotropic mode which we observed in regime I.

Fundamentally, the two circular modes induced by inertial mass can be understood in terms of the additional spin degree of freedom at the skyrmion boundary. If we look at the spin structure at the circular boundary of the skyrmion (i.e., the boundary DW), two circular modes correspond to the excited spin waves which propagate in the opposite directions inside the DW channel^2^. One is a slow spin wave propagating along the CCW direction, and the other is a fast spin wave propagating along the CW direction. For the slow spin wave, the spins adiabatically align with the direction of the DW, thus it is insensitive to the inertia (corresponding to the low-lying gyrotropic mode).

For the fast spin wave, on the other hand, the spin oscillation is out of phase with the direction of the DW, therefore a strong inertial effect emerges (see Fig. 3d). This implies that the spin wave excitation in the DW channel is necessary to observe the inertial effect. This consideration allows us to understand why the strong inertial effect is only observed with the oscillating SHST, and not the oscillating magnetic field. The magnetic field gives a global torque to the boundary DW, so that the entire spin exhibits a coherent motion, which leads to a breathing motion. The SHST-induced torque, on the other hand, depends on the magnetization direction, so that it exerts a non-uniform torque to the boundary magnetization, which can trigger the spin wave excitation in the DW channel. We note that an unusual drop is observed at the end of region III, as shown in Fig. 2a,b. We think that this is due to the confined geometry effect since the *R*_max_ and *ΔS* increase rapidly and the disk size starts to affect the skyrmion motion.

## Discussion

**Figure 4:**
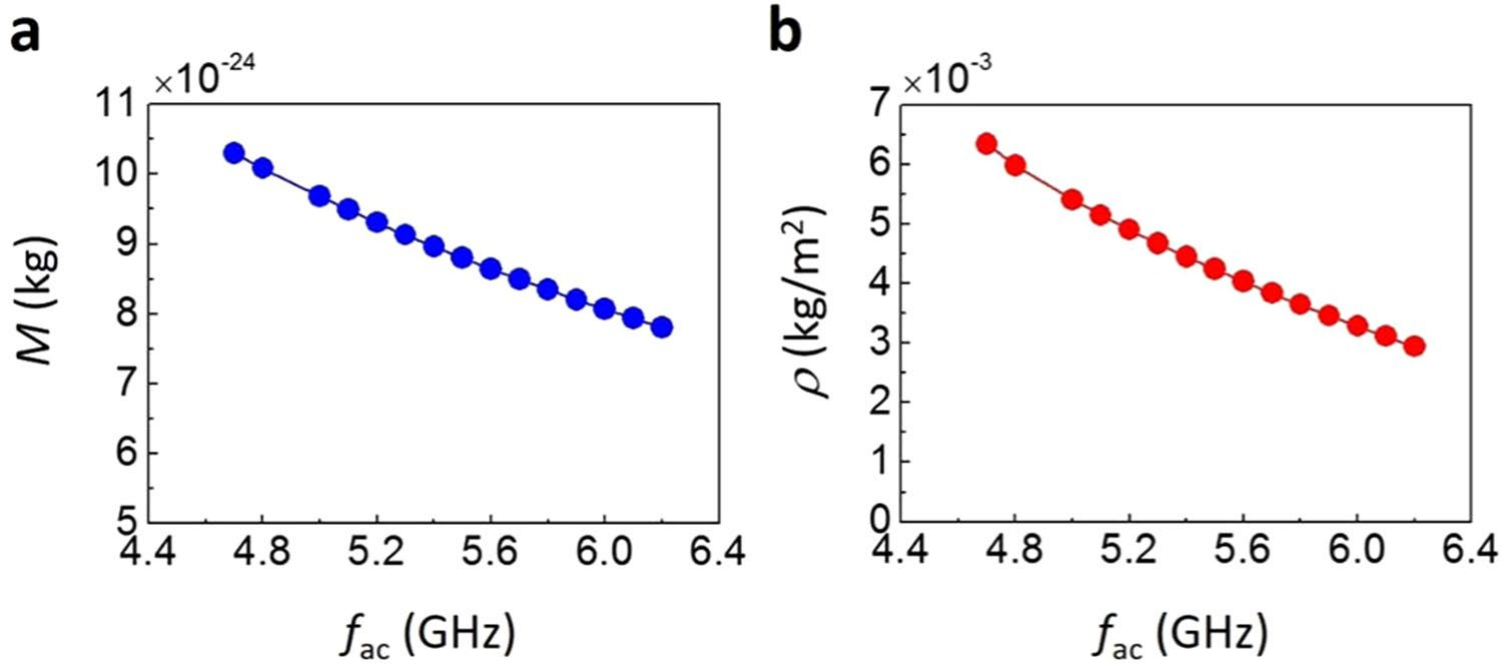
Inertial mass and mass density of a skyrmion in regime III. (**a**,**b**) Inertial mass *M* (**a**) and mass density *ρ* ≡ *M/S*_mean_ (**b**) as a function of oscillating frequency *f*_ac_. Here, *S*_mean_ is the time mean value of *S* during the steady-state inertia-driven resonant excitation motion.

The inertia-driven resonant excitation of the skyrmion in regime III provides a way of estimating the inertial mass. According to Eq. (3), the inertial mass can be calculated from the sum of the frequencies of the two circular modes: *M* = − *G*/ (${\omega }_{+}$ + ${\omega }_{-}$). In Fig. 4, we summarize the inertial mass and mass density as a function of the frequency ${f}_{{\rm{ac}}}$. Up to this point, the inertial effect has only been observed in the relaxation process. Thus, it is not easy to extract the inertial mass because one needs to extract both frequencies during the short relaxation process. However, our results provide a more convenient way to determine the inertial mass because one of the frequencies (the higher frequency) is fixed by the external oscillating source. Furthermore, the inertial effect appears constant during the resonant excitation, which is greatly beneficial to the experimental approaches. We note that to consider more realistic situations, further study will need to take into account the thermal-fluctuation effects. However, the key finding in our work would still hold even at finite temperature, because the zero temperature model can well describe the inertia-driven skyrmion motion at finite temperature^2,24^.

In conclusion, we found that the inertial effect of a skyrmion can be observed by applying an oscillating SHST. The intriguing observation can be attributed to the additional degree of freedom at the boundary of the skyrmion. Because the SHST exerts a non-uniform torque on the boundary spin of the skyrmion, the spin waves are excited and propagate along the boundary, which forms an inertia-driven hypocycloidal trajectory. Our result also provides a convenient way to estimate the inertial mass of the skyrmion, which is crucial to understanding the motion of the skyrmion. This work therefore paves the way for the development of skyrmion-based spintronic devices.

## Methods

The micromagnetic simulations were performed using the OOMMF code^30^ with the DMI extension^31^. In a continuum model, the interfacial DMI energy density can be expressed as^7,21^

5 $${\varepsilon }_{{\rm{D}}{\rm{M}}}=D\,[{{\bf{e}}}_{y}\cdot ({\bf{m}}\times \frac{{\rm{\partial }}{\bf{m}}}{{\rm{\partial }}x})-{{\bf{e}}}_{x}\cdot ({\bf{m}}\times \frac{{\rm{\partial }}{\bf{m}}}{{\rm{\partial }}y})],$$

where **m** = [*m*_*x*_(*t*, *x*, *y*), *m*_*y*_(*t*, *x*, *y*), *m*_*z*_(*t*, *x*, *y*)] is the dimensionless unit vector of the local magnetization in the FM layer, and *D* [J/m^2^] is the DMI energy density constant. The material parameters were set as follows: the saturation magnetization *M*_s_ = 1.13 × 10^6^ A/m, the exchange stiffness constant *A* = 1.6 × 10^−11^ J/m, the DMI constant *D* = 3.0 × 10^−3^ J/m^2^, the perpendicular magnetocrystalline anisotropy energy *K*_u_ = 1.28 × 10^6^ J/m^3^, and the damping constant *α* = 0.015. The mesh size was set to 1.0 × 1.0 × 0.6 nm^3^, and the interpolation of magnetic configuration in the (*x*, *y*) space was used. Note that the magnetostatic exchange length and the magnetocrystalline exchange length in this system are $\sqrt{2A/{\mu }_{0}{M}_{{\rm{s}}}^{2}}\approx$ 4.75 nm and $\sqrt{A/{K}_{{\rm{u}}}}\approx$ 3.54 nm, respectively. Our calculation was conducted at *T* = 0, where thermal fluctuation is absent.

We adopted a model system of a thin FM disk with a diameter of 80 nm on a HM electrode; e.g., Pt(HM)/Co(FM), which induces broken inversion symmetry in the FM disk through the spin-orbit interaction with the bottom HM layer (see Fig. 1a). To realize steady motion of the skyrmion through excitation, an alternating current (AC) at various frequencies was applied along the HM electrode layer to induce an oscillating SHE, which exerts a SHST to the FM layer.

The governing equation of the simulation is the Landau-Lifshitz-Gilbert equation with the time-varying SHST term. It is described as^32^

6 $$\frac{\partial {\bf{m}}}{\partial t}=-\gamma \,{\bf{m}}\times {{\bf{H}}}_{{\rm{eff}}}+\alpha \,{\bf{m}}\times \frac{\partial {\bf{m}}}{\partial t}+\gamma {H}_{{\rm{SH}}}\,\sin (2\pi {f}_{{\rm{ac}}}t)\,{\bf{m}}\times ({\bf{m}}\times {{\bf{e}}}_{y}),$$

where *γ* is the gyromagnetic ratio; *f*_ac_ is the AC frequency; and **H**_eff_ is the effective magnetic field stemming from the exchange, the anisotropy, the demagnetization and the DMI energy terms. The third term on the right side of equation (6) is the time-varying SHST term given the assumption that the AC is applied along the in-plane *x* direction. The coefficient *H*_SH_ is a time-independent parameter which is defined as^32,33^

7 $${H}_{{\rm{S}}{\rm{H}}}=\frac{{\mu }_{{\rm{B}}}{\theta }_{{\rm{S}}{\rm{H}}}\,{j}_{{\rm{c}}}}{\gamma e{M}_{{\rm{s}}}{t}_{z}},$$

where *θ*_SH_ is the spin Hall angle of the adjacent HM layer, *μ*_B_ is the Bohr magneton, *e* is the electron charge, *t*_*z*_ is the thickness of the FM layer (*t*_*z*_ = 0.6 nm in our simulations), and *j*_c_ is the AC amplitude which flows in the *x* direction. It was also assumed that most electrons flowed through the HM under layer (with a thickness typically of 3~5 nm), as the thickness and the conductivity of the HM layer can be much greater than those of the FM layer^33^. In this study, therefore, we only considered the SHST and neglected spin-transfer torques as well as the Rashba effect^32,33^. Throughout this study, the amplitude of the SHST was set to *H*_SH_ = 0.1 × 10^4^ A/m for the MHz-frequency and *H*_SH_ = 2.69 × 10^4^ A/m for the GHz-frequency excitations.

In our nanodisk system, the skyrmion has a Neel-type domain wall (a hedgehog-type skyrmion), and the skyrmion topological number is ${N}_{{\rm{sk}}}=(1/4\pi ){\iint }^{}{\bf{m}}\cdot ({\partial }_{x}{\bf{m}}\times {\partial }_{y}{\bf{m}})dxdy=-1$. The area, *S*, and the position of the skyrmion, (*X*, *Y*), were defined by the area of the region where the out-of-plane magnetization component *m*_*z*_ is greater than 0 and by the centre of the area, respectively (see Supplementary note 1). Note that the radial position of the skyrmion is *R* = (*X* ^2^ + *Y* ^2^)^1/2^ (see Fig. 1b). The frequency spectra were obtained using the fast Fourier transform. See Supplementary note 2 for detailed information about the simulation method.

## Electronic supplementary material

Supplementary information

Supplementary Video

Supplementary Video

Supplementary Video
