# Supplementary material for: Inertia-driven resonant excitation of a magnetic skyrmion
Source: Sci Rep. 2017 Oct 25;7:13993. doi: 10.1038/s41598-017-13241-2 (PMC5656687; doi:10.1038/s41598-017-13241-2)
Supplement: Supplementary file 1 — Supplementary information [file 41598_2017_13241_MOESM1_ESM.pdf]

Supplementary Information

Inertia-driven resonant excitation of a magnetic skyrmion

Takayuki Shiino[1], Kab-Jin Kim[2], Ki-Suk Lee[3*] and Byong-Guk Park[1*]

[1]*Department of Materials Science and Engineering, KAIST, Daejeon, 34141, Republic of Korea*

[2]*Department of Physics, KAIST, Daejeon 34141, Republic of Korea.*

[3]*School of Materials Science and Engineering, Ulsan National Institute of Science and Technology, Ulsan 689-798, Republic of Korea*

Supplementary Note 1. Definition of the skyrmion position in a nanodisk

Here, we show the definition of the position of a skyrmion that is used in this study. In Supplementary Figure S1, each bar inside the boundary of the skyrmion represents the chain of unit calculation cells along the *x* and *y* directions, respectively. In this study, we defined the skyrmion position **R** = (*X*, *Y*) as follows;

$$X = \frac{1}{N}\sum_{i}^{N} x_i , \qquad Y = \frac{1}{N}\sum_{i}^{N} y_i ,$$

where *N* is the number of the bars inside the boundary of the skyrmion, x_i (y_i) represents the center position value of the i-th bar. In our simulation, the shape of the skyrmion was kept almost circular even in excited states under an oscillating spin-Hall-spin torque (SHST). Therefore, this definition approximately gives the centre position of the skyrmion.

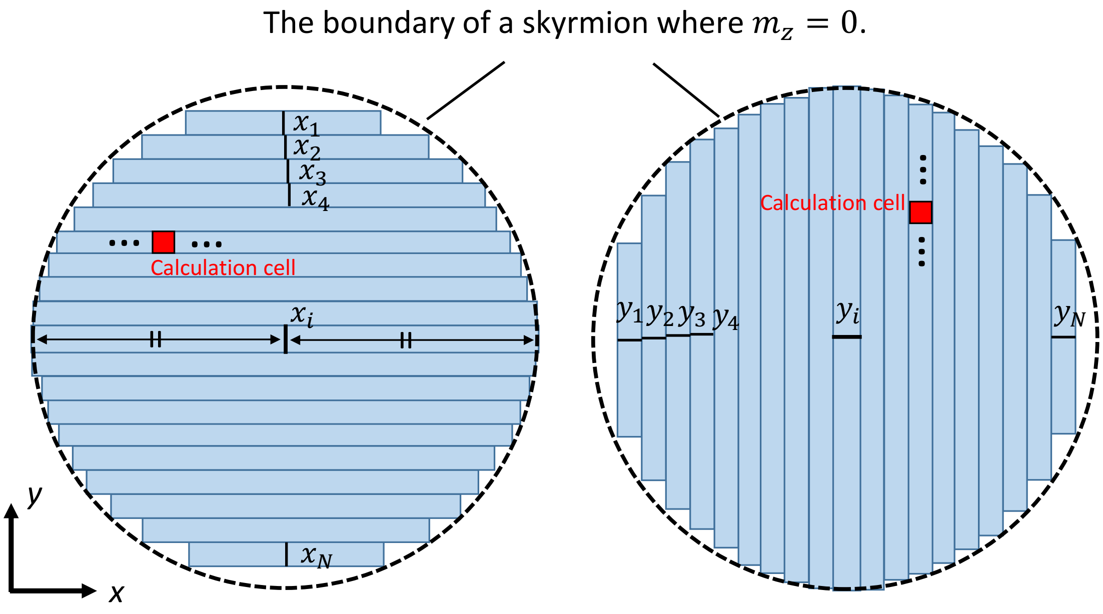

Supplementary Figure S1 | Definition of the position of a skyrmion.

Supplementary Note 2. Additional information about the simulation method

In this section, we note additional information about our simulation method. In terms of the driving force, i.e. the SHST, we used small amplitude of $H_{\mathrm{SH}} = 0.1\times10^4$ A/m for the low-frequency range (0.1~0.9 GHz), and large amplitude of $H_{\mathrm{SH}} = 2.69\times10^4$ A/m for the high-frequency range (1~9 GHz) for the following reasons. First, in the low-frequency range, the oscillating SHST with large amplitude drives the skyrmion further away, and it reaches the disk edge resulting in the destruction of the skyrmion. Second, in the high-frequency range, we observed that the oscillating SHST with small amplitude could not induce strong inertial effects in regime III (see Fig.2 in the main text). Also, it should be noted that we used a small damping constant of $\alpha = 0.015$ compared to that of thin ferromagnetic films[1], but a small damping constant has been reported for CoFeB thin film samples so that the damping constant of $\alpha = 0.015$ should not be unrealistic[2–4].

In our simulations, the skyrmion has a Neel-type domain wall (a hedgehog-type skyrmion) due to the interfacial Dzyaloshinskii-Moriya interaction (DMI). In this case, the skyrmion can be driven along the *y* direction, whereas current is applied along the *x* direction. It can be explained by Thiele's collective coordinate equations of motion for a hedgehog-type skyrmion[5]. Supplementary Figure S2**a** shows a snapshot image of the skyrmion in an equilibrium state, and the cross-section magnetization configuration along the green dot line is shown in Supplementary Figure S2**b**. At the edge boundary, the magnetization is tilted from the z direction because of the interfacial DMI[6].

The total calculation time was 300~500 ns for each simulation. The steady-state oscillation data during 100~300 ns (300~500 ns for 6.2 GHz excitation case) were used to obtain frequency spectra by means of the fast Fourier transform.

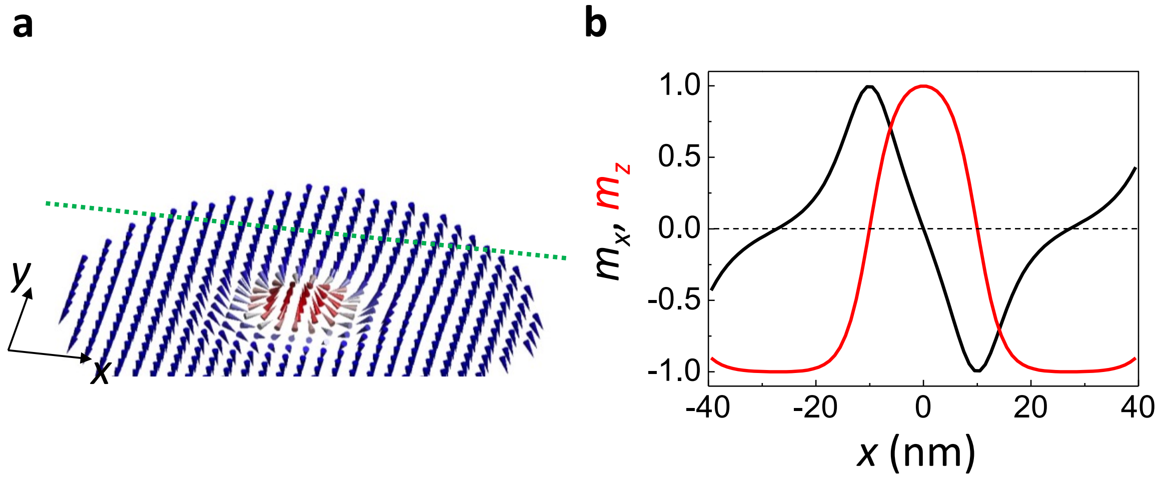

Supplementary Figure S2 | Magnetization configuration of a skyrmion in the equilibrium state. a, The schematic of the magnetization configuration of the skyrmion in a equilibrium state. The color indicates the magnitude of m_z: red for $m_z = +1$ and blue for $m_z = -1$. **b**, The cross section profile along the green dot line in **a**. The x (black) and z (red) components of magnetization are shown.

Supplementary Movies

Supplementary Movie 1 shows the steady-state low-lying gyrotropic motion of the skyrmion induced by the oscillating SHST with 0.26 GHz.

Supplementary Movie 2 shows the steady-state breathing motion of the skyrmion induced by the oscillating SHST with 2.8 GHz.

Supplementary Movie 3 shows the steady-state dual excitation of the gyrotropic and the breathing modes induced by the oscillating SHST with 6.0 GHz.

Each movie shows the steady-state excitation motion of the skyrmion during 250-260 ns.

References

1. Tserkovnyak, Y., Brataas, A. & Bauer, G. E. W. Enhanced Gilbert Damping in Thin Ferromagnetic Films. *Phys. Rev. Lett.* **88,** 117601 (2002).

2. Sampaio, J., Cros, V., Rohart, S., Thiaville, A. & Fert, A. Nucleation, stability and current-induced motion of isolated magnetic skyrmions in nanostructures. *Nat. Nanotechnol.* **8,** 839–844 (2013).

3. Iihama, S. *et al.* Gilbert damping constants of Ta/CoFeB/MgO(Ta) thin films measured by optical detection of precessional magnetization dynamics. *Phys. Rev. B* **89,** 174416 (2014).

4. Liu, X., Zhang, W., Carter, M. J. & Xiao, G. Ferromagnetic resonance and damping properties of CoFeB thin films as free layers in MgO-based magnetic tunnel junctions. *J. Appl. Phys.* **110,** 33910 (2011).

5. Tomasello, R. *et al.* A strategy for the design of skyrmion racetrack memories. *Sci. Rep.* **4,** 6784 (2015).

6. Rohart, S. & Thiaville, A. Skyrmion confinement in ultrathin film nanostructures in the presence of Dzyaloshinskii-Moriya interaction. *Phys. Rev. B* **88,** 184422 (2013).
